# Perspectives on the Role of Professional Laboratory Animal Science Associations

**DOI:** 10.3390/bs7040073

**Published:** 2017-10-23

**Authors:** Ann Tourigny Turner, Ekaterina Botovchenco Rivera, Cynthia Pekow

**Affiliations:** 1 American Association for Laboratory Animal Science (AALAS), 9190 Crestwyn Hills Drive, Memphis, TN 38125-8538, USA; ann.turner@aalas.org; 2 Brazilian Association for Laboratory Animal Science (SBCAL), Av. Prof. Dr. Orlando de Paiva, 87, Cidade Universitária, Sao Paulo, CEP 05508-270, Brazil; botovchencorivera@gmail.com; 3 International Council for Laboratory Animal Science (ICLAS), Rue Washington, 1050 Brussels, Belgium

The three authors of this letter had the honor and pleasure of being invited to the international meeting “Laboratory Animal Science, Moving Forward Together: State of the Art in Non-Clinical Models for Neurodegenerative Diseases”, held in Varadero, Cuba, 21–24 June 2017. Individually, we represent the American Association for Laboratory Animal Science (AALAS), the Brazilian Association for Laboratory Animal Science (SBCAL), and the International Council for Laboratory Animal Science (ICLAS). In this brief communication, we highlight the role of professional associations in advancing laboratory animal science, with specific examples from our participation in this meeting in Cuba.

## 1. AALAS

Dr. Ann Turner, Executive Director of AALAS, spoke to the group about the structure, purposes, and roles of associations. An association is a group of individuals who voluntarily enter into an agreement to form an organization to accomplish a purpose. There are four major types of associations: trade associations; professional societies; philanthropic associations; and fraternal/social/civic groups. Laboratory animal science associations are most commonly professional associations. However, one very unique aspect of associations in laboratory animal science is that they often include as members not only the professionals working with research animals (researchers, veterinary and animal care staff, managers), but also trade professionals (vendors of relevant products and services such as cage wash equipment or animal production), as well as regulatory authorities (agents of government or accrediting bodies). Such a diverse membership provides advantages in communication and networking among the different stakeholders in the community. At this meeting, for example, representatives from a company that manufactures animal housing provided a non-commercial session on understanding how ventilated rodent caging contains allergens and infectious agents and improves air quality for the animals.

Volunteers are the backbone of associations, relied on for policy-making and oversight, and, in some cases, they are also the workforce. In laboratory animal science, larger associations such as AALAS rely on trained professional staff to provide specialized services including governance, education, publications, communications, finance, information technology, human resources, and database management. The professional staff implements policy, programs, and services at the direction of a governing body.

Associations work to advance their missions. AALAS’ mission statement establishes AALAS as an association of professionals that advances responsible laboratory animal care and use to benefit people and animals. AALAS’ core values include the belief that the use of laboratory animals in scientific and medical research is essential to the improvement and protection of the quality of all life. Furthermore, humane and responsible care of laboratory animals is vital to quality research and, as such, an essential aspect of AALAS’ endeavors. AALAS is dedicated to building and disseminating a knowledge base in laboratory animal science for the education and training of those who work in this field.

Associations such as AALAS advance their mission through various means including: information dissemination (publications, conferences); self-regulation (certification, accreditation, standards of practice, code of ethics); professional development (education, training, products, publications); public relations (influence public opinion, advocacy); coalition building (common good); scientific endeavors (research, position statements); and government relations (lobbying, advocacy). At this meeting in Cuba, for example, Dr. Turner provided two oral presentations related to understanding associations, and distributed some professional development products (texts, training materials) to interested meeting attendees.

AALAS has a substantive education and training component including the development and dissemination of training manuals and materials, on-line learning programs, and other training and reference materials. AALAS certifies laboratory personnel at three levels for technicians and one management certification. The Cuban Association for Laboratory Animal Science (SCCAL) discussed their strong need to develop a professional certification program for their members, not only for personal development and improved technical knowledge and skills, but also to increase the visibility and respect of animal care staff as key members of the research team. AALAS’ experience of over 50 years in developing and sustaining certification programs provides an example of how associations can share expertise. As SCCAL works to develop and implement a certification program, connections with AALAS and other associations that have similar programs can be of great assistance in determining options and factors to consider, as well as pitfalls to avoid.

The annual AALAS National Meeting is the largest gathering of the laboratory animal science community in the world. The hallmark of a professional association is networking, and AALAS provides the opportunity for collegial exchange at many levels, including the local level, through branches, and the national/international level, through conferences and community platforms. In a similar vein, time spent at both the formal and informal sessions at this gathering in Varadero was key to strengthening the network among our colleagues.

## 2. SBCAL

The Brazilian Association for Laboratory Animal Science (SBCAL) is another national group that was invited to participate in the Varadero congress. Dr. Ekaterina Rivera, President of SBCAL, provided a keynote address on considerations of animal quality.

Dr. Rivera pointed out how ethical, legal, economic, scientific, biotechnological, and animal welfare considerations affect research animal quality. Poor animal quality may lead to animal suffering and distress, consequently affecting welfare and the results of the studies. Good animal quality cannot exist if institutions do not assume their responsibilities. In addition, the facility veterinarian has a key role in the process of assuring animal quality. Different types of quality systems were briefly described.

Brazil has recently implemented, for the first time, national legislation governing the use of research animals, including the requirement for formal ethical oversight of this work. In separate conversations, the experience of creating and implementing legislation as well as developing training programs and materials for the people involved was shared. The meeting participants from Cuba actively participated, asking many questions about ethical committees, as well as requesting suggestions for initiating and writing legislation to govern the use of animals used in experiments; this is another example of the important interchange afforded at professional congresses.

## 3. ICLAS

Working with national associations and their federations, a number of international laboratory animal science organizations have roles in the promotion of animal welfare and quality programs of animal care and use. Dr. Cynthia Pekow, Secretary General of ICLAS, provided a discussion on ICLAS and its role. Originating in 1956, ICLAS is a not-for-profit association headquartered in Belgium. ICLAS members include stakeholders in laboratory animal science, including national associations, scientific societies, and unions; international federations; and scientific institutions and organizations. Together, the members form an international network to promote basic harmonization of standards for laboratory animal science, with a priority of encouraging quality animal-based science in developing nations. Through ICLAS, members have a voice in respected international science unions and agencies such as the World Organization for Animal Health (OIE), the International Council of Scientific Unions (ICSU), the Council of International Organizations of Medical Science (CIOMS), and the United Nations (UNESCO). An example of a successful outcome of these collaborations is the 2012 publication of the CIOMS-ICLAS International Guiding Principles for Biomedical Research Involving Animals. Harmonization and promotion of standards of laboratory animal quality, care, and ethical consideration benefit the ability to collaborate internationally, and improve confidence in accepting science protocols conducted across nations.

Research facilities and laboratories participating in the ICLAS Laboratory Animal Quality Network (LAQN) and Performance Evaluation Program (PEP) are directly assisted in validating the microbiological and genetic quality of their research animals. The network includes collaborative laboratories across the globe. Any nation, such as Cuba, that produces animals for research can take advantage of the LAQN to facilitate animal quality assurance activities.

Of direct value to participants at the Varadero congress, ICLAS was able to share some training resources in Spanish to aid personnel in recognizing common medical conditions in laboratory rodents. In addition, information on grants available for educational opportunities in laboratory animal science was discussed. Connections were made for contacts with research staff working with nonhuman primates on other Caribbean islands, to exchange information on enrichment protocols and management practices.

## 4. Conclusions

Prior to the start of the international meeting in Varadero, Drs. Pekow and Rivera met for a day with members of the Cuban Association for Laboratory Animal Science. The meeting included a workshop on considerations for animal care, including the enrichment of the animal housing environments, and a discussion of training of personnel. Members of SCCAL presented summaries of the work at their research institutions, and the challenges they face. Such direct interactions provided an opportunity for sharing and personal connection, to permit the exploration of the challenges, differences, and similarities in laboratory animal science that are encountered in each nation.

Meetings such as the State of the Art in Non-Clinical Models for Neurodegenerative Diseases provide a forum for the exchange of scientific information. However, for the attendees, there is an additional intangible aspect—one of the most important reasons for participating in these congresses—the relationships that are forged and strengthened through interpersonal interactions can be key to opening windows for opportunities to grow, learn, and share. Discussions that began in formal question and answer sessions at the talks and poster sessions continued at mealtimes, and continue still, via email, internet, and other modes of communication. These meetings can be the impetus for planning future educational events, such as a workshop on how to develop a certification program, now planned for an upcoming international meeting in Brazil in 2018.

